# Influence of Trp flipping on carbohydrate binding in lectins. An example on *Aleuria aurantia* lectin AAL

**DOI:** 10.1371/journal.pone.0189375

**Published:** 2017-12-12

**Authors:** Josef Houser, Stanislav Kozmon, Deepti Mishra, Sushil K. Mishra, Patrick R. Romano, Michaela Wimmerová, Jaroslav Koča

**Affiliations:** 1 CEITEC MU - Central European Institute of Technology, Masaryk University, Brno, Czech Republic; 2 National Centre for Biomolecular Research, Faculty of Science, Masaryk University, Brno, Czech Republic; 3 Institute of Chemistry, Slovak Academy of Sciences, Bratislava, Slovak Republic; 4 Structural Glycobiology Team, Systems Glycobiology Research Group, RIKEN Global Research Cluster, Wako, Saitama, Japan; 5 Baruch S. Blumberg Institute, Doylestown, Pennsylvania, United States of America; Wake Forest University, UNITED STATES

## Abstract

Protein–carbohydrate interactions are very often mediated by the stacking CH–π interactions involving the side chains of aromatic amino acids such as tryptophan (Trp), tyrosine (Tyr) or phenylalanine (Phe). Especially suitable for stacking is the Trp residue. Analysis of the PDB database shows Trp stacking for 265 carbohydrate or carbohydrate like ligands in 5 208 Trp containing motives. An appropriate model system to study such an interaction is the AAL lectin family where the stacking interactions play a crucial role and are thought to be a driving force for carbohydrate binding. In this study we present data showing a novel finding in the stacking interaction of the AAL Trp side chain with the carbohydrate. High resolution X-ray structure of the AAL lectin from *Aleuria aurantia* with α-methyl-l-fucoside ligand shows two possible Trp side chain conformations with the same occupation in electron density. The in silico data shows that the conformation of the Trp side chain does not influence the interaction energy despite the fact that each conformation creates interactions with different carbohydrate CH groups. Moreover, the PDB data search shows that the conformations are almost equally distributed across all Trp–carbohydrate complexes, which would suggest no substantial preference for one conformation over another.

## Introduction

Lectins are carbohydrate-binding proteins that are widely used in medicinal research for lectin-staining of cells and tissues as well as for glycoprotein analysis. They are also a promising tool for targeted drug development. One of the predominantly used lectins is AAL from *Aleuria aurantia*—the first fungal lectin with a solved 3D structure.[1] It combines high affinity towards fucose and fucosylated oligosaccharides with an ability to recognize core fucosylated oligosaccharides with α1–6 linked fucose.[2, 3] This moiety has remarkable importance in the analysis of changes in the protein glycosylation and consequently in the diagnosis of cancer and other cell-surface related investigations. There are five slightly different binding sites per AAL monomer and there is evidence for the presence of at least one so called high-affinity binding site.[2] However, it was published, that the high-affinity binding site and the core-fucose binding site are at two distinct parts of the molecule.[3] Recent studies of AAL homologues further support the evidence of variable binding site composition and affinities within the lectin family.[4–7] Therefore, the current investigation aims to delineate the molecular basis of sugar preferences and affinity enhancement.

Aromatic residues are well known for mediating the π-π interaction between proteins and their ligands including nucleic acids, aromatic ligands and other proteins.[8–10] Moreover, the interaction between an aromatic residue and a non-polar group (e.g. methyl), so called CH-π interaction, were found to be important driving forces in biomolecular interactions.[11–13] Tryptophan (Trp) is the most frequently found amino acid involved in this process, however, tyrosine, phenylalanine and histidine may also form CH-π interactions.[14] We have recently demonstrated the strength of non-polar CH-π interaction in lectin-sugar binding using the lectin RSL from *Ralstonia solanacearum*, a member of AAL lectin family.[15, 16] In the PDB database, the two main relative orientations of the Trp side chain can be found in Trp stacking complexes; however, the effect of these conformations on the strength of the stacking interactions have not been fully characterized. In this study, we present the high resolution X-ray structure of the AALN224Q lectin complex with α-methyl-l-fucoside where the Trp side chain is found in both conformations. Interestingly, both Trp conformations are visible in electron density with equal occupancy. Given these observations we focused on the analysis of the role that these two Trp conformations have on orientating the CH-π interaction in AAL ligand binding. Protein-carbohydrate interactions involving CH-π interaction may be studied by direct experimental biophysical methods such as fluorescence spectroscopy[17], isothermal titration calorimetry[16] or nuclear magnetic resonance[12] or various *in silico* methods including molecular docking and quantum chemical calculations.[13] As the present system is too complicated (16 Trp residues per monomer, 8 of them involved in 5 different binding sites) to directly apply biophysical techniques, we have used computational chemistry methods to evaluate the energy criteria for both Trp conformations in lectin–carbohydrate complex and also bioinformatics tools to reveal statistical importance of the Trp residue conformation phenomenon.

## Material and methods

α-methyl-l-fucoside (αMeFuc) was purchased from Interchim, Montluçon, France, Basic chemicals were purchased from Sigma-Aldrich, St.Louis, USA, Duchefa, Haarlem, Netherlands and Applichem, Darmstadt, Germany.

### Protein expression and purification

Recombinant lectin AALN224Q was prepared as described previously.[3] Briefly, *Escherichia coli* BL21 Star(DE3) (Invitrogen) cells transformed with the pQE-AALN224Q vector were cultivated according to the manufacturer’s protocol. Cells were harvested by centrifugation and lysed in phosphate buffer saline (PBS) using an Avestin C5 homogenizer. His-tagged AALN224Q was isolated from the protein extract by affinity chromatography on IMAC HisTrap HP (GE Healthcare) using 50mM Na_2_HPO_4_, 1M NaCl, pH 7.0 as loading buffer. Elution was performed using an imidazole step gradient (250-800mM imidazole) in loading buffer. Fractions containing pure AALN224Q protein were pooled, transferred to PBS and used for crystallization.

### Crystallization and X-ray diffraction data collection

Purified protein was subsequently used for crystallization experiments using the hanging drop method. The protein was concentrated to 5 mg/ml, αMeFuc added to 2mM final concentration and the solution was mixed with precipitant (12% PEG 6K, 120 mM citrate, pH 5.0) in 2:1 and 1:1 ratio, respectively. Plates were incubated at 17°C until crystals were formed. Crystals were cryo-cooled at 100K after soaking for the shortest possible time in reservoir solution supplemented with 20% (*v/v*) glycerol. The X-ray diffraction experiments were performed at BESSY II in Berlin, Germany on the 14.1 beamline.

### Structure determination

Collected diffraction images were processed using XDS[18] and converted to structure factors using the program package CCP4 version 6.1[19] with 5% of data reserved for R_free_ calculation. The structure of the complex was determined using the molecular replacement method with Molrep 11.0[20] with the structure of AAL/Fuc (1OFZ)[1] without the ligands as the starting model. Refinement of the molecule was performed using Refmac5[21] alternated with manual model building in Coot 0.7[22]. Sugar residues and other compounds present were placed manually using Coot. Water molecules were added by Coot and checked manually. The addition of alternative conformations where necessary resulted in a final structure that was validated by the wwPDB validation server (http://www.pdb.org) and deposited in the PDB Database with accession number 5MXC.

### QM—Interaction energies calculation

#### Computational details

**Fig 1:**
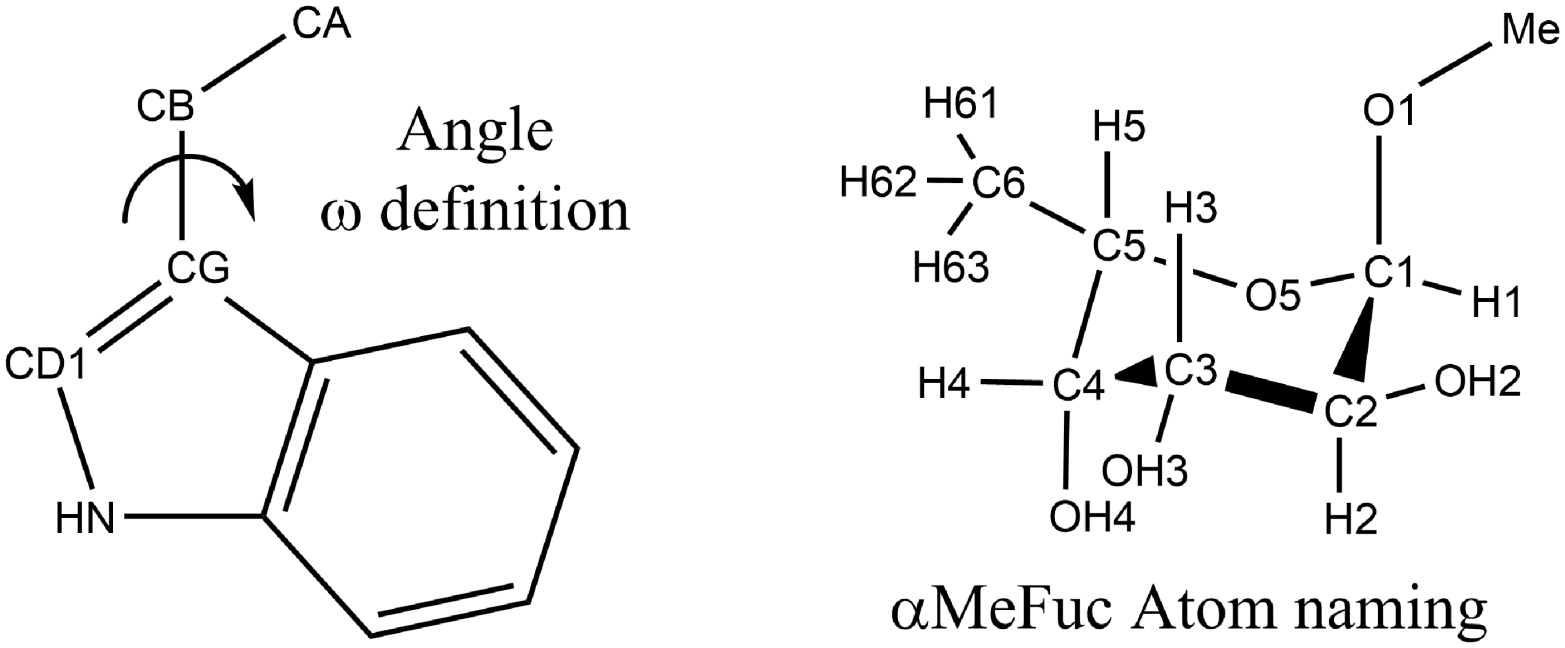
Schematic representation of the ω angle definition and the atom naming in the αMeFuc residue.

The crystal structure of the fucose binding lectin AAL N224Q mutant (PDB ID 5MXC) from *Aleuria aurantia* served as a template for all used binding site models. The structure contains a monomeric unit of the lectin bound to α-methyl-l-fucoside residue (αMeFuc) in all five binding sites. All five binding sites are very similar and the main difference among them is that three of them contain the Trp residue which creates CH-π stacking interaction with bound Fuc residue, whereas the remaining two binding sites contain the Tyr residue which also creates CH-π interaction. Interestingly, the crystal structure also showed that Trp in two out of three such binding sites can accommodate two different conformations. The binding site models were prepared for all the binding sites. Moreover, models with two possible Trp conformations were prepared for the binding sites where this phenomenon was observed. To see the difference between the Trp containing sites, the Trp194 flipped conformation was prepared artificially. Each binding site model contains all amino acid residues side chains up to C-alpha carbon, which interacts with bound fucose molecule. Binding sites models are named consecutively from the AAL N-terminal as previously published[1] and the name contains the name of the stacking amino acid. The *Site01_Tyr* model include αMeFuc, Trp15, Arg24, Glu36, Gln38, Ile74, Ile76, **Tyr92**, Trp97; *Site02_Trp* includes αMeFuc, Trp68, Arg77, Glu89, Val91, Gly100, Gln101, Pro128, Ile130, **Trp149**, Trp153; *Site03_Trp* includes αMeFuc, Trp120, Arg131, Glu146, Val148, Gly156, Ala157, Gly176, Leu178, **Trp194**, Trp199; *Site04_Tyr* includes αMeFuc, Ile173, Arg177, Arg179, Glu191, Cys193, Tyr200, Gly202, Gly203, Pro223, Ile225, **Tyr241**, Trp245; and *Site05_Trp* includes αMeFuc, Trp219, Arg226, Glu238, Ala240, Ile274, Ile276, **Trp292**, Trp298 (S1 Fig). The conformation of the stacking tryptophan residue is described by angle ω, which is defined by atoms CA-CB-CG-CD1 (atom names are based on PDB database nomenclature). Based on the angle ω conformations of flipped Trp residue side chain approximately correspond to the *gauche(+)* and *gauche(-)* regions. The ω angle definition and αMeFuc atom naming is shown in Fig 1.

The geometric structure of all prepared AAL binding site models was optimized. The alpha carbons of all amino acid residues were fixed to their crystallographic positions during the optimization, and the rest of the model was fully optimized without any restraints or constraints. The geometry optimization was done employing the Density Functional Theory with Grimmes’s empirical corrections to the dispersion energy (DFT-D3) with Becke-Johnson damping function.[23] The Becke-Perdew functional[24, 25] with triple-ζ quality basis set def2-TZVPP implemented in the TURBOMOLE program package was used. All calculations were performed in the TURBOMOLE 7.0 program package[26, 27] employing the resolution of identity for DFT calculation algorithm[28–30] (ri-dft routine in TURBOMOLE package). The interaction energies for all optimized models were calculated with the basis set super position error correction[31, 32] as is implemented in the TURBOMOLE program at the same level of theory.

### MD (tryptophan flipping)

The structure of free (no ligand) and bound (αMeFuc present in all five binding sites) N224Q AAL mutant lectin were prepared and solvated in a rectangular box of TIP3P water molecules extending 11Å away from the edges of the solute(s) using tLeap. The protein and glycan were described with the Amber ff14SB[33] and GLYCAM06[34] (version 06j-1) force fields, respectively. The simulation systems were equilibrated by first performing 3000 steps of energy minimization to relax unfavorable conformations, followed by 300 ps NPT simulation to equilibrate solvent density (see S1 File for detailed equilibration protocol). The final snapshot was used as starting structure for subsequent umbrella sampling calculations. All the molecular dynamics (MD) simulations were carried out using AMBER14 suite[35] of programs. All of the umbrella sampling simulations were performed using the final structure obtained from multi-step equilibration protocol.

The conformation of Trp in Site2 (Trp149), Site3 (Trp194) and Site5 (Trp292) along the dihedral angle was sampled in both free and bound states of the lectin. In this study, the dihedral angle along the CB–CG bond of Trp (i.e., CA–CB–CG–CD2) is termed as the reaction coordinate (χ). The whole range (-180 to +180) was divided into 89 windows, each window separated by 4 degrees from each other along the reaction coordinate. Starting conformations for each window were generated by a 100ps NPT constrained dynamics simulation where the dihedral angle was changed slowly to a specified value set for each window using in-house tool PMFLib.[36] This was followed by a 500 ps equilibration at 300 K where a force constant of 200 kcal.mol^-1^.rad^-2^ was used to restrain dihedral angle specified for each window. A harmonic biasing potential, Vb(χ), is added to the total energy to enhance the sampling of conformational space near to target value of the dihedral angle to that window. A 5 ns NPT production run at 300 K was performed for each window. The collective variable was collected at each 200 fs. Periodic boundary conditions are used with a 9Å atom-based cut-off distance for the non-bonded interactions. Long-range electrostatic interactions were handled using a reaction field and the medium dielectric constant was set to 78.3. The temperature was regulated by Langevin dynamics with the collision frequency 0.5. No bond length constraints were applied. Long-range electrostatic behavior was controlled with the particle mesh Ewald (PME) method. All the production simulations were carried out on GPU machines using pmemd (cuda) code of AMBER14.

#### Umbrella sampling simulations

The PMF W(χ), or the change in free energy along the coordinate χ, can be defined as:

$$W(\chi )=-k_{B}Tln\langle \rho (\chi )\rangle$$

where (χ) is the Boltzmann weighted average. The separate 89 simulations were then combined to obtain the unbiased average distribution function F(χ) and its associated potential of mean force (PMF). The weighted histogram analysis method (WHAM) approach is used to obtain average F(χ). A memory efficient WHAM software package (v. 2.0.9) by Grossfield[37] was used for getting unbiased umbrella sampling distributions and PMF at various times during the simulations. PMF calculation was done using 360° periodicity, 89 windows, with the reaction coordinates ranging from -180 to 180 and number of padding values set to be 0. Convergence tolerance was set to be 0.01. The bootstrapping error analysis was performed by computing averages from a set of N points chosen at random. Statistical uncertainties were calculated as standard deviation of these averages by repeating this procedure 100 times.

## Results and discussion

### AALN224Q structure

The high resolution structure of AALN224Q co-crystallized with αMeFuc was solved by molecular replacement using the protein coordinates of chain A of the native AAL structure (1OFZ) as the search model (Table 1).

**Table 1:** Data collection and refinement statistics for AALN224Q complex with αMeFuc. Data in parentheses for highest resolution shell.

*Data collection*	AALN224Q/αMeFuc
Beamline, diffraction source	14.1, BESSY II
Wavelength (Å)	0.9180
Space group	C2
a, b, c (Å)	132.25; 48.63; 57.58
α, β, γ (°)	90.00, 103.19, 90.00
No. of monomers in asymmetric unit	1
Resolution range (Å)	64.38–1.14 (1.17–1.14)
Total no. of reflections	534413 (73069)
No. of unique reflections	130546 (18870)
Completeness (%)	99.9 (99.5)
Redundancy	4.1 (3.9)
〈 I/σ(I)〉	13.0 (4.8)
R_merge_ (%)	0.066 (0.265)
CC(1/2)	0.998 (0.926)
Wilson B (Å^2^)	5.0
*Refinement statistics*	
No. of amino acids	313
No. of protein atoms	2662
No. of solvent atoms	492
No. of ligand atoms	128
Resolution limits	64.38–1.14 (1.17–1.14)
No. of reflections in working set	123974 (8972)
No. of reflections in test set	6572 (534)
Final R_cryst_ (%)	0.138 (0.190)
Final R_free_ (%)	0.146 (0.182)
Mean B factor (Å^2^)	7.5
R.m.s. deviations	
Bonds (Å)	0.008
Angles (°)	1.450
Planar groups (Å)	0.008
Chiral volumes (Å^3^)	0.087
Ramachandran plot	
Most favoured (%)	98.0
Allowed (%)	2.0
Outliers (%)	0.0

**Fig 2:**
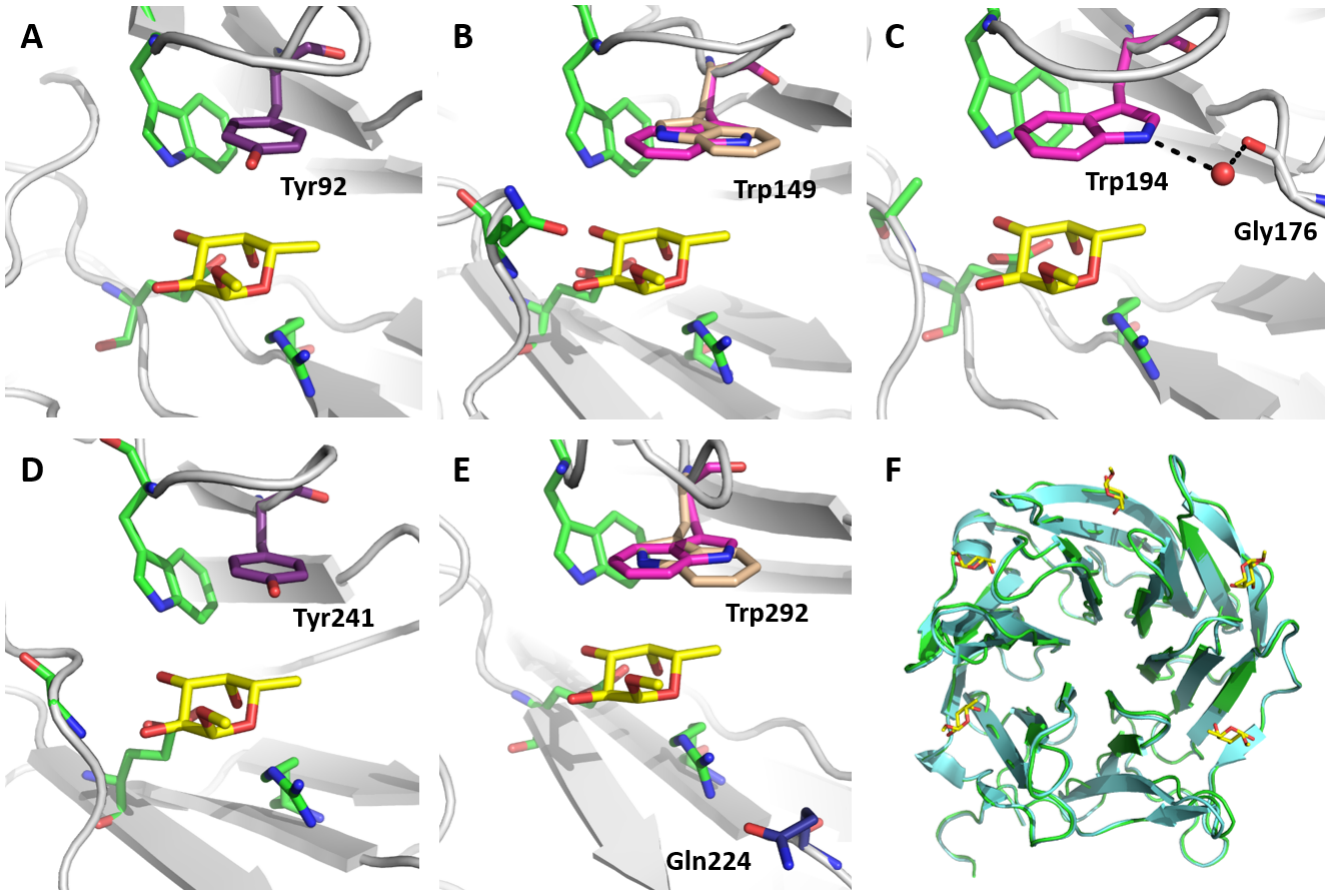
AAL N224Q binding sites. (A)-(E) individual binding sites 1 to 5. Colour scheme: αMeFuc—yellow, stacking Tyr—violet, stacking Trp g(-)–purple, stacking Trp g(+)–pink, mutated Asn224Gln—dark blue, bridging water molecule in site 3 shown as red sphere. (F) Comparison of AAL N224Q with αMeFuc ligands (green, yellow) and chain A of AAL PDB: 1OFZ (cyan).

The protein adopts the 6-bladed β-propeller fold (Fig 2F) highly similar to a previously determined structure of the AAL/Fuc complex.[1] No significant variations of the backbone conformation were observed between the chains of the AAL N224Q complex and previously determined structures,[1, 38] with RMSD varying from 0.186 to 0.249 Å. Comparison of the AAL structures shows that all binding sites are rigid and do not change the structure upon the binding and so do not suggest possible cooperativity between the binding sites. The single point mutation N224Q reported previously to affect the binding affinity of site 5[3] is not directly involved in ligand binding (Fig 2E). Additional organic molecules (glycerol) originating from cryo-protecting solution were detected. Glycerol molecules are coordinated in the vicinity of the ligand in binding Site1 and Site5, respectively. However, this does not alter the ligand position compared to previously determined AAL structure complex with Fuc.[1]

As the high resolution structure allowed for a precise atom placement, the residues responsible for ligand binding were reanalysed. The orientation of all side chains involved in ligand recognition by the lectin is identical to the previously published structure (1OFZ) with the exception of CH-π interacting tryptophan residues in binding Site2 (Trp149) and Site5 (Trp292). For each of these two sites, high resolution electron density revealed the presence of two Trp conformations in app. 50:50 occupancy ratio, while the single position of the ligand is kept with 100% occupancy. This phenomenon has not been described before for any structure of homologous lectins, even though both conformers were observed in a particular site for different chains or different complexes of one lectin (Table 2 and S1 Table).

**Table 2:** Analysis of CH-π stacking Trp conformations in lectins from AAL family (from structures deposited in PDB).

Protein, PDB ID	Site1	Site2	Site3	Site4	Site5	Site6
AAL N224Q* 5mxc	Tyr	*g(+)* and *g(*‒*)*	*g(*‒*)*	Tyr	*g(+)* and *g(*‒*)*	NP
**AAL** 1ofz, 1iub, 1iuc	Tyr	*g(+)* or *g(*‒*)*	*g(*‒*)*	Tyr	*g(*‒*)*	NP
**AFL** 4agi, 4agt, 4aha, 4ah4, 4c1y, 4uou, 4d52, 4d4u	Tyr	*g(*‒*)* or *g(+)*	Tyr	*g(*‒*)*	*g(*+*)*	Tyr
**RSL and mutated RSL**^†^ 2bs5, 2bs6, 2bt9, 3zi8, 5ajb, 5ajc, 4i6s, 4csd	*g(*‒*)*	*g(*‒*)*	*g(*‒*)*	*g(*‒*)*	*g(*‒*)*	*g(*‒*)*
**BambL**^†^ 3zw0, 3zwe, 3zzv, 3zw2, 3zw1	*g(*‒*)*	Tyr	*g(*‒*)*	Tyr	*g(*‒*)*	Tyr

* this study, Tyr—stacking tyrosine instead of tryptophan residue present, NP—binding site not present.

^**†**^six binding sites are formed by oligomerization. Sites 3 and 5 correspond to Site1; Sites 4 and 6 correspond to Site2

Based on CA-CB-CG-CD1 torsion angle ω, we label these conformations as *gauche(+)* and *gauche(‒)* or *g(+)* and *g(*‒*)*, respectively. In binding Site3, where CH-π interaction is also mediated by tryptophan residue (Trp194), only *g(*‒*)* conformation was found. This is stabilized by a water molecule bridge between NE1 of Trp194 and the backbone O of Gly176. Regardless the tryptophan conformation, there is only one preferred orientation of αMeFuc in all sites (Fig 2). The hydrogen bond network of the αMeFuc is not affected by the Trp conformation within any of the binding sites.

### PDB database data mining

Based on obtained Trp conformations in the AAL N224Q X-ray structure, we examined the PDB database for the Trp-carbohydrate complexes where we focused on the Trp side chain conformation found in these complexes. The PDB database was searched to find all binding sites with sugar ligands that are bound by CH-π stacking interaction with tryptophan. We used PatternQuery program for searching.[39] The sugar ligand was defined as a ligand that contains a five or six membered ring with single bonds only, which contains one oxygen atom and four or five carbon atoms, and have a OH group bound to the ring carbon corresponding to C3 or C4 in carbohydrate nomenclature. Criteria for the stacking were defined based on the distance and angle between the Trp side chain and the carbohydrate ring (more details in S2 File). The CH-π stacking interaction was defined using the distance between the aromatic centre of the Trp residue and the closest CH atom of the ligand. PDB search resulted in 265 carbohydrate or carbohydrate like ligands (based on PDB residue names) in 5 208 Trp containing motives found in 2 036 PDB structures. The Trp side chain ω angle varies from -176 to 179°, and represents the whole conformational space. Splitting the values based on basic conformations to *eclipsed* (0 ±30°), *gauche (+)* (90 ±60°), *gauche (-)* (-90 ±60°) and *trans* (180 ±30°) shows distributions with two major peaks in *g(+)* and *g(-)* areas (S2 Table). The *trans* conformation was derived from the conformer counts of 18 structures (0.35%). The *eclipsed* conformation contains 581 complexes (11.15%). The rest of the complexes can be found in *g(+)* (2526 structures, 48.50%) or *g(-)* (2 083 structures, 40.00%) conformations. The PDB data search shows an almost equal distribution of the Trp side chain conformation across all carbohydrate CH–π complexes. Obtained data suggest no preference for Trp side chain dihedral angle.

### Interaction energies of stacking Trp

**Fig 3:**
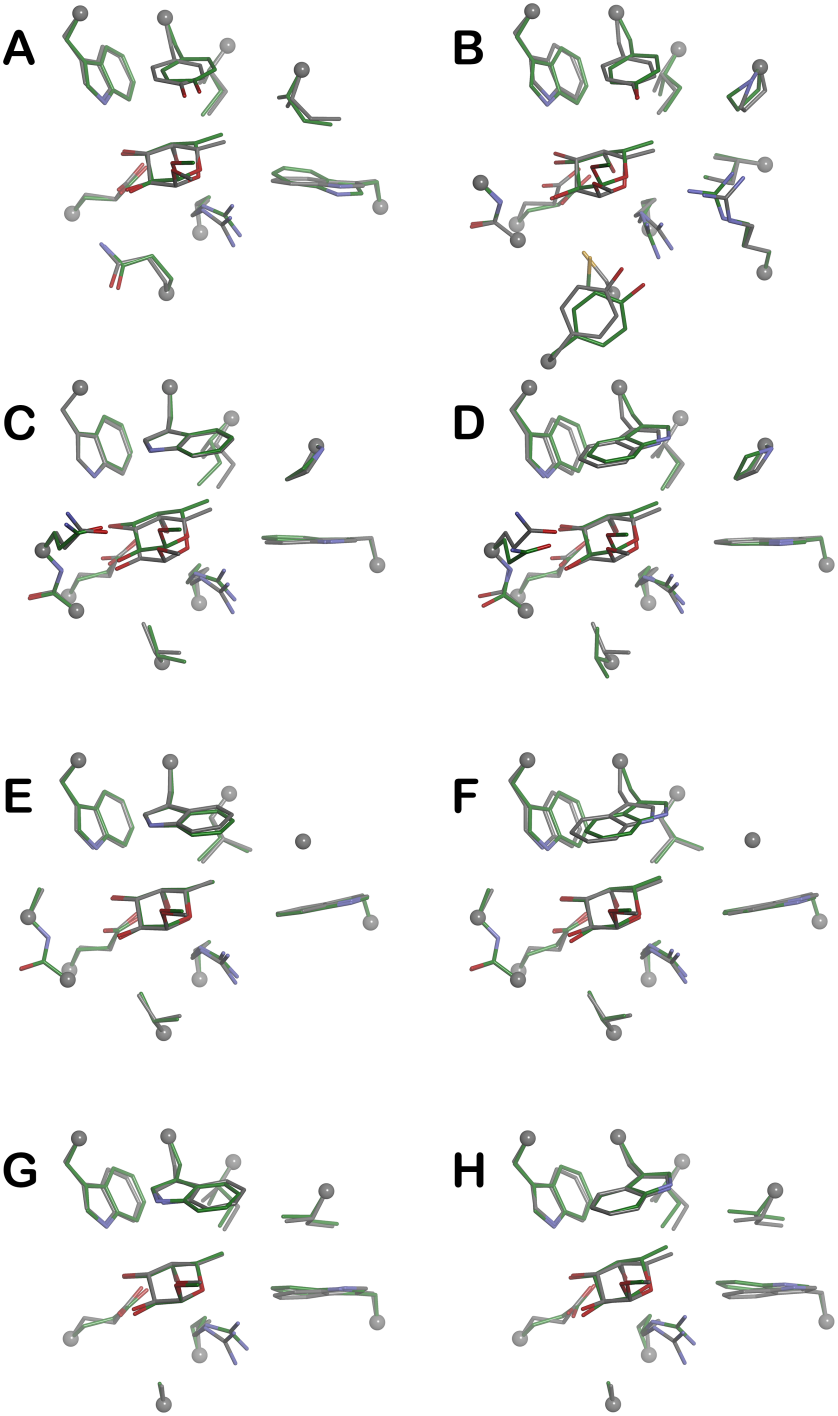
Optimized binding site models. Superimposition of the optimized binding site models (green carbon atoms) with crystallographic binding site structures (grey carbon atoms) of Site1 (**A**), Site4 (**B**), Site2 (**C** *g(+)*; **D** *g(-)*), Site3 (**E** *g(+)*; **F** *g(-)*) and Site5 (**G** *g(+)*; **H** *g(-)*), respectively.

All binding site models were optimized and optimized structures were compared to the crystallographic positions. To compare the overall similarity of the optimized model with the original X-ray structure we have calculated RMSD values for all heavy atoms (Table 3). Calculated RMSD values lie in the range of 0.166 to 0.540 Å. In general, observed values show that the binding site models overcome only slight changes during the optimization and are very similar to the crystallographic positions. Overlay of the structures can be seen in Fig 3. The biggest changes in the binding site geometry can be seen in the *Site04_Tyr* and *Site02_Trp g(-)* models with RMSD 0.540 and 0.499 Å, respectively. In the case of *Site04_Tyr* model the biggest changes in the structure were observed for the residues lying under the αMeFuc residue Cys193, Tyr200 or Arg177 (Fig 3B).

**Table 3:** The RMSD values and the distances in Å between the αMeFuc hydrogens included in the CH-π stacking interaction with aromatic amino acid side chain.

Binding Site	Stacking AA	Conf.	RMSD [Å]	Ring Centroid	αMeFuc Hydrogen Atom				
					H3	H4	H5	H61	H62
Site1	Tyr92		0.369	Ph	4.15	3.28	3.03	2.99	4.07
Site2	Trp149	*g(+)*	0.394	Ph	5.07	4.53	3.11	3.13	3.18
				Pyrr	3.85	3.20	2.86	3.11	4.04
	Trp149	*g(-)*	0.499	Ph	3.75	3.52	2.57	3.38	3.97
				Pyrr	5.34	4.14	3.56	2.68	3.52
Site3	Trp194	*g(+)*^(a)^	0.167^(b)^	Ph	4.69	4.07	2.90	2.90	3.37
				Pyrr	3.75	3.04	3.11	3.38	4.53
	Trp194	*g(-)*	0.355	Ph	3.43	3.33	2.55	3.47	4.17
				Pyrr	5.00	3.84	3.39	2.61	3.59
Site4	Tyr241		0.540	Ph	4.97	3.89	3.57	2.93	3.83
Site5	Trp292	*g(+)*	0.316	Ph	4.67	4.20	2.87	3.04	3.37
				Pyrr	3.79	3.16	3.10	3.39	4.48
Site5	Trp292	*g(-)*	0.409	Ph	3.45	3.39	2.48	3.47	4.10
				Pyrr	5.01	3.89	3.32	2.59	3.49

^(a)^ Artificial model.

^(b)^ RMSD to the starting structure.

The biggest movement in *Site02_Trp g(-)* model was observed for the Gln101 side chain (Fig 3D). All other models show only very small difference to the starting structure with RMSD up to 0.4 Å. Based on that we can conclude that optimized structures are in good agreement with the crystallographic structures.

Within all binding sites, common hydrogen bond interactions of the αMeFuc OH3 hydroxyl group with Glu and Trp, and OH4 group with Glu and Arg, and ring oxygen O5 with Arg side chain can be found (Fig 3). To describe the hydrogen atoms responsible for the strongest CH-π interaction, we measured the distances between the H3—H61/2 hydrogens and the centroid of the phenyl part of the Tyr or Trp stacking residue. Additionally, we also measured these distances with the centroid on the pyrrole part of the indole side chain of the Trp residue (Table 3). Our previous studies show that the CH-π interaction is strongest where the distance between the hydrogen atom and the ring centroid is in the range 2.3 to 2.5 Å (4.0–5.4 kcal/mol), still very strong in range 2.5–3.0 Å (approx. 3.5 kcal/mol) and still attractive in range 3.0–3.5 Å (approx. 2.0 kcal/mol).[40, 41] Analysis of the measured distances shows that in the tyrosine binding sites *Site01_Tyr* and *Site04_Tyr*, up to three CH-π interactions are possible. The hydrogen atoms H3, H4 and H61 are involved in the stacking and distances are in a range from 2.9 to 3.3 Å where one is shorter than 3.0 Å. It suggests that the H61 atom has stronger interaction compare to H4 and H5 in *Site01_Tyr* model. However, in the case of *Site04_Tyr* model only two interactions with distances up to the 3.5 Å were found. Similarly, the H61 atom has a stronger interaction within a distance of 2.9 Å and a weaker interaction for the H5 atom with a distance of 3.5 Å. The H3 atom does not show stacking interaction with Tyr241 and was found 3.8 Å away from ring centre. However, we should note that this observation can be caused by slightly bigger movements in the binding site during the geometry optimization and the stacking interaction within the mentioned distance is still attractive but much less than in the optimal distance. In the case of Trp binding sites *Site02_Trp*, *Site03_Trp* and *Site05_Trp*, measured distances identify mainly six possible CH-π dispersion interactions in each binding site. However, in *Site03_Trp* and *Site05_Trp* with *g(-)* conformation one more interaction for H3 atom with distance of 3.4 Å was observed. The distances range from 2.5 to 3.5 Å. Similar behavior can be seen in all Trp binding site models. Each binding site contains two strong interactions with H–ring centroid distance less than 3.0 Å and four distances between 3.0–3.5 Å. However, in the case of *Site02_Trp* and *Site05_Trp* in *g(+)* conformations, only one interaction closer than 3.0 Å can be seen, and make this Trp binding site slightly weaker compare to other Trp sites (Table 4). Measured distances reveal that the strongest interaction has H5 and H61 hydrogen atoms across all Trp binding sites. The H5 atom predominantly interacts with the phenyl part of the Trp residue in all models, whereas the H61atom interacts with phenyl part only in *g(+)* conformation of the Trp side chain. When we compare the binding sites with the different Trp conformation, there is no difference in a number of CH-π interactions, however there is a difference in the hydrogen atoms interactions and with various atoms in the Trp indole side chain. In the binding site models with the *g(+)* conformation H4, H5, H61 and H62 hydrogen atoms are involved in the CH-π interactions, whereas in the models with *g(-)* conformation the H3, H4 H5 and H61 atoms are involved. Optimized structures also show that in the binding sites with Trp *g(+)* conformation the H5, H61 and H62 atoms interact mainly with phenyl part, whereas the pyrrole part interacts mainly with H4 and H5 and only partially with H61. The situation in the binding sites with *g(-)* conformations is slightly different. In this case, the atoms H3, H4 and H5 interact predominantly with the phenyl structure, H61 interacts partially with phenyl structure but exhibits a stronger interaction with the pyrrole structure, which H5 only partially interacts with.

**Table 4:** Interaction energies (kcal/mol) of the αMeFuc with stacking residue.

	Binding Site Model							
	*Site01_Tyr*	*Site02_Trp*		*Site03_Trp*		*Site04_Tyr*	*Site05_Trp*	
Conformation		*g(+)*	*g(-)*	*g(+)*^(a)^	*g(-)*		*g(+)*	*g(-)*
E_int_ Exp.	-7.31^(b)^							
E_int_ αMeFuc –Trp/Tyr	-5.30	-6.96	-7.59	-6.97	-7.77	-4.59	-7.10	-7.96

^(a)^Artificial model.

^(b)^Value is calculated from K_d_ measured in Ref [42] using formula ΔG = R·T·ln(K_d_).

For all the binding site models, the CH-π interaction energy between the αMeFuc and stacking Tyr or Trp residue was calculated. Interaction energy (E_int_) was calculated on the optimized model structures where only the αMeFuc and stacking residue was used for the energy evaluation. We use the basis set superposition error (BSSE) correction for the energy evaluation. The calculated E_int_ are summarized in Table 4. Values of the E_int_ clearly show the difference between the Tyr and Trp binding sites. The CH-π E_int_ with the Tyr is in the range -4.6 –-5.3 kcal/mol; whereas in the case of the Trp the interaction is much stronger with an E_int_ between -7.0 –-8.0 kcal/mol. Experimental interaction energy ΔG of the αMeFuc to AAL is -7.31 kcal/mol (calculated based on measured K_d_). However, this value represents an average E_int_ across all AAL binding sites and does not distinguish between two types of binding site (Tyr or Trp). Interestingly, the measured value is very close to the E_int_ calculated for the αMeFuc–Trp interaction. Calculated values of E_int_ correspond with the number of possible CH-π interaction based on measured H-centroid distances. The Tyr residue with less possible CH-π interactions has weaker dispersion interaction. When we compare the influence of the flipping on the E_int_ we can see that there is almost no difference in the calculated interaction energy. The difference between the *g(+)* and *g(-)* conformations is less than 0.8 kcal/mol, which is on the edge of the DFT-D method accuracy. The observed E_int_ suggest that both stacking Trp conformations are equivalent in strength even though they create slightly different interactions with αMeFuc. This is also supported by the equivalent occupation of both conformations in the experimentally measured X-ray density.

### Dynamics of Trp flipping

**Fig 4:**
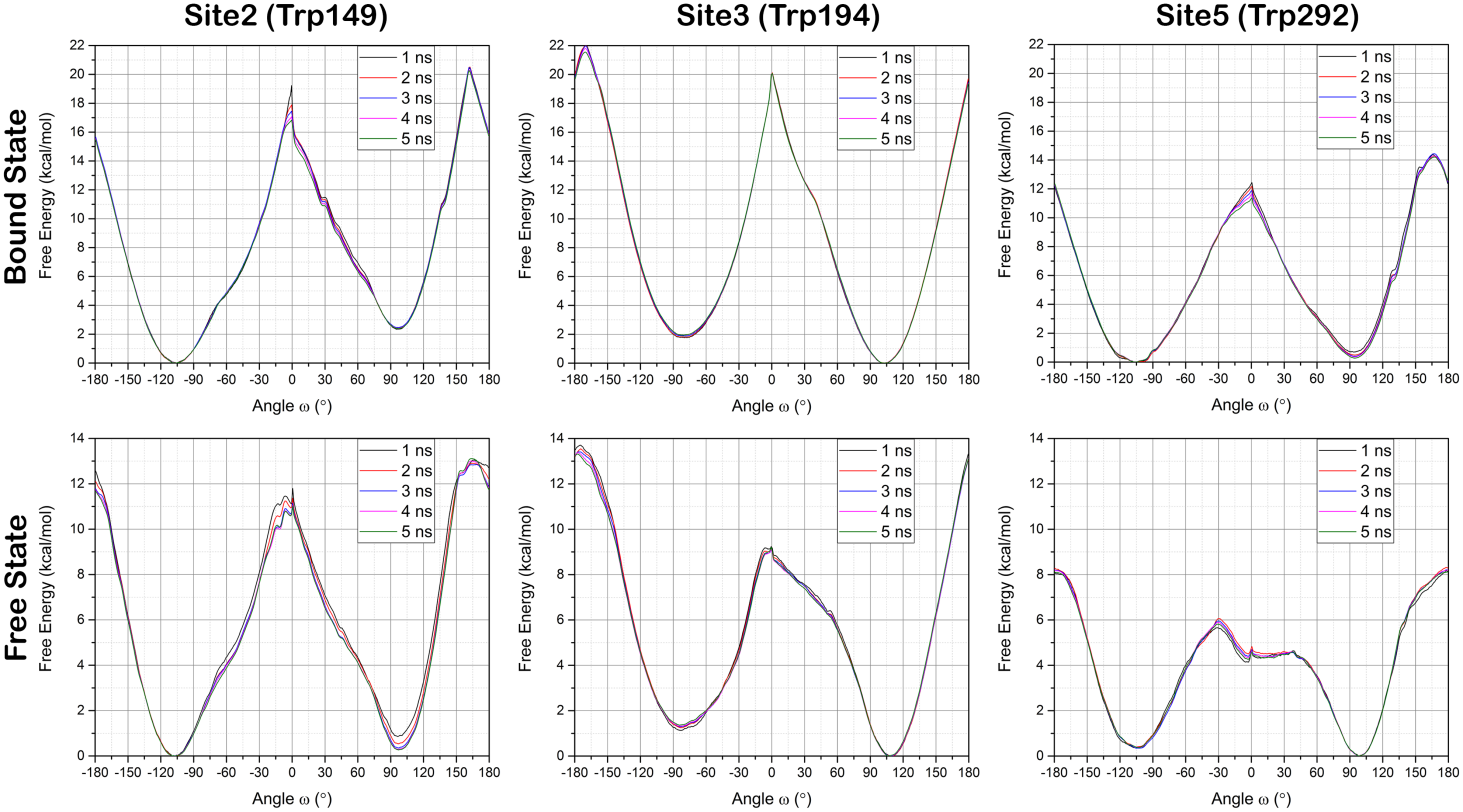
Tryptophan flipping energy plots. Umbrella sampling simulation results for the tryptophan (Trp149, Trp194, Trp292) flipping in free and bound states for total sampling time of 1, 2, 3, 4, and 5 ns. Potential of mean force (PMF) results or tryptophan flipping as a function of its CA-CB-CG-CD1 dihedral (ω).

The presence of two Trp conformation in the binding sites also raise the questions of whether the observed conformational change (flipping) is energetically favorable, what is the energetic barrier to flipping, and what consequence flipping might have on αMeFuc binding. To investigate the dynamics of the Trp flipping we employed Umbrella Sampling molecular dynamic simulations. We investigated orientation of the Trp in Site2, 3 and 5 of the N224Q mutant. A series of MD simulations combined with umbrella sampling provides the change in the free energy of the system along the reaction coordinate. We obtained the change in the relative free energy of the system between two conformations of the Trp, free energy barrier between these two conformations of the Trp residue and the possible direction of flipping between these two states. We calculated these values for Trp in Site2, 3 and 5 in the presence (bound) and absence (free) of the αMeFuc residue. In each case, 89 MD simulations, starting from a dihedral angle separated by 4 degrees from each other (89 umbrella windows) were setup and MD was extended up to 5 ns for each window. Histograms of the collective variables for all umbrella sampling windows was used to ensure a sufficient overlap between adjacent windows (S4–S9 Figs). PMFs generated at each 1 ns time span during 5ns simulations shows their evolution and excellent convergence with the 5ns simulation. Fig 4 shows that simulations are well converged.

PMF calculations of models with free binding site show a free energy barrier of about 11.0, 9.2 and 5.8 kcal/mol between two possible conformations of Trp149, Trp194, and Trp292 respectively. Whereas, the barrier is much higher when the ligand is present in the binding site and range from 11.4 up to 20 kcal/mol (Fig 4). In bound state, the Trp194 (Site3) has the highest transition barrier, ~20 kcal/mol, compared to Trp149 and Trp292 whose two conformations are seen in the crystal structure. The direction of flipping from *g(+)* conformation (ω = ~98°) to *g(-)* is preferred in the anticlockwise direction through the *eclipsed* conformation (S2 and S3 Figs). Interestingly, Trp194 shows that there is a difference of 1.3 kcal/mol and 1.9 kcal/mol between *g(+)* and *g(-)* conformations in free and bound states respectively. However, in the other two cases, this difference in free state is below 0.5 kcal/mol, which is well below the computational errors in such a calculation. Observed differences in stable minima can also be a result of slight changes in the lectin structure during the dihedral angle driving. This could result from the structures occupying slightly different energy minima on the potential energy surface. On the other hand, the above-mentioned difference may due to the fact that Trp194 prefers just one conformation, while for Trp149 and Trp292 both conformations have almost equivalent energies and both conformations are observed. However, the preference of one Trp194 conformation in Site3 can be a result of the interaction with the water molecule (Fig 2C), which can stabilize only one preferred conformation. Since the strength of CH-π interactions with αMeFuc for both the conformations is same, it can be rationalized that if both *g(+)* and *g(-)* are of equivalent energy in the free state then both conformations of Trp are possible.

## Conclusions

Analysing the high resolution structure of *Aleuria aurantia* lectin AAL in complex with αMeFuc, we identified CH–π interacting Trp residue being present in two conformations with the same occupancy in binding Site2 and Site5, while only one conformation is preferred in Site3. The ligand position was not affected by the Trp conformation when compared to previously published AAL/Fuc structure.[1, 38] To our knowledge, this phenomenon has never been reported previously for any known lectin–sugar or, in general, protein–carbohydrate complex.

We have done a PDB database search for the Trp conformations in the Trp–carbohydrate complexes. We have found over 5200 motives of the carbohydrate-Trp complexes with 256 carbohydrate or carbohydrate like structures. Observed motives show that Trp can be found in both, *g(+)* or *g(-)*, conformations with almost equal distribution 48.50% (2 526 motives) or 40.00% (2 083 motives) for *g(+)* or *g(-)*, respectively. Based on the above finding, we employed the *in silico* approach to further analysis the Trp-αMeFuc interaction in the binding sites of the AAL lectin. The optimized binding site models show that there is only a slight difference in the behavior between the flipped conformations. In both conformations, the αMeFuc residue creates six possible CH–π interactions and the difference in the E_int_ between the conformers is less than 0.8 kcal/mol which is negligible. The main difference is only in the αMeFuc hydrogen atoms, which are involved in the CH–π dispersion interaction. The *in silico* calculations, where no difference in interaction energy between the different Trp conformers was obtained, support observed the almost equal distribution of the Trp conformers within the found carbohydrate-Trp binding motives from PDB database.

The fact that Trp residues can mediate sugar–lectin interactions has been known for some time; however, the possible equality in switched Trp conformations for sugar ligand stabilization was neither observed nor proven before. This phenomenon brings even higher variability into AAL family sugar binding patterns and may possibly play a role in the different binding site affinities reported previously.[2, 3] It might be taken into account when analyzing the lectin-sugar interaction, explaining the experimental thermodynamic binding parameters or designing the binding site during sugar-interacting proteins engineering.

## Supporting information

S1 Fig AAL binding sites. Definition of the AAL binding site models. Fixed CA are shown as a balls.
(TIF)

S2 Fig The Trp149 interactions. Trp149 interaction with αMeFuc in the binding Site2 (A). Umbrella sampling starting orientations of Trp149 in ligand bound state (B).
(TIF)

S3 Fig The Trp149 orientations. Orientation of Trp149 along the CA-CB-CG-CD2 dihedral angle.
(TIF)

S4 Fig The umbrella sampling histograms for the Trp149 (free). The number of counts (x-axis) for the umbrella sampling histogram at each Ø values in Trp149 (free) state flipping simulation. The overlapped Gaussian-shape histograms confirm the full sampling of whole space -180 to 180 degree.
(PNG)

S5 Fig The umbrella sampling histograms for the Trp149 (bound). The number of counts (x-axis) for the umbrella sampling histogram at each Ø values in Trp149 (bound) state flipping simulation. The overlapped Gaussian-shape histograms confirm the full sampling of whole space -180 to 180 degree.
(PNG)

S6 Fig The umbrella sampling histograms for the Trp194 (free). The number of counts (x-axis) for the umbrella sampling histogram at each Ø values in Trp194 (free) state flipping simulation. The overlapped Gaussian-shape histograms confirm the full sampling of whole space -180 to 180 degree.
(PNG)

S7 Fig The umbrella sampling histograms for the Trp194 (bound). The number of counts (x-axis) for the umbrella sampling histogram at each Ø values in Trp194 (bound) flipping simulations. The overlapped Gaussian-shape histograms confirm the full sampling of whole space -180 to 180 degree.
(PNG)

S8 Fig The umbrella sampling histograms for the Trp292 (free). The number of counts (x-axis) for the umbrella sampling histogram at each Ø values in Trp292 (free) flipping simulations. The overlapped Gaussian-shape histograms confirm the full sampling of whole space -180 to 180 degree.
(PNG)

S9 Fig The umbrella sampling histograms for the Trp92 (bound). The number of counts (x-axis) for the umbrella sampling histogram at each Ø values in Trp292 (bound) state flipping simulation. The overlapped Gaussian-shape histograms confirm the full sampling of whole space -180 to 180 degree.
(PNG)

S1 Table CH-π stacking Trp conformation in lectins from AAL family. (PDF)

S2 Table Distribution of the Trp conformation in observed carbohydrate—Trp complexes from PDB database. (PDF)

S1 File Equilibration protocol for umbrella sampling MD. Detailed information about used protocol for Umbrella Sampling MD.
(PDF)

S2 File PDB database data mining details. Description of the methodology used for the PDB data mining.
(PDF)
