# Supplementary figures and images for: Influence of Trp flipping on carbohydrate binding in lectins. An example on Aleuria aurantia lectin AAL
Source: PLoS One. 2017 Dec 12;12(12):e0189375. doi: 10.1371/journal.pone.0189375 (PMC5726637; doi:10.1371/journal.pone.0189375)

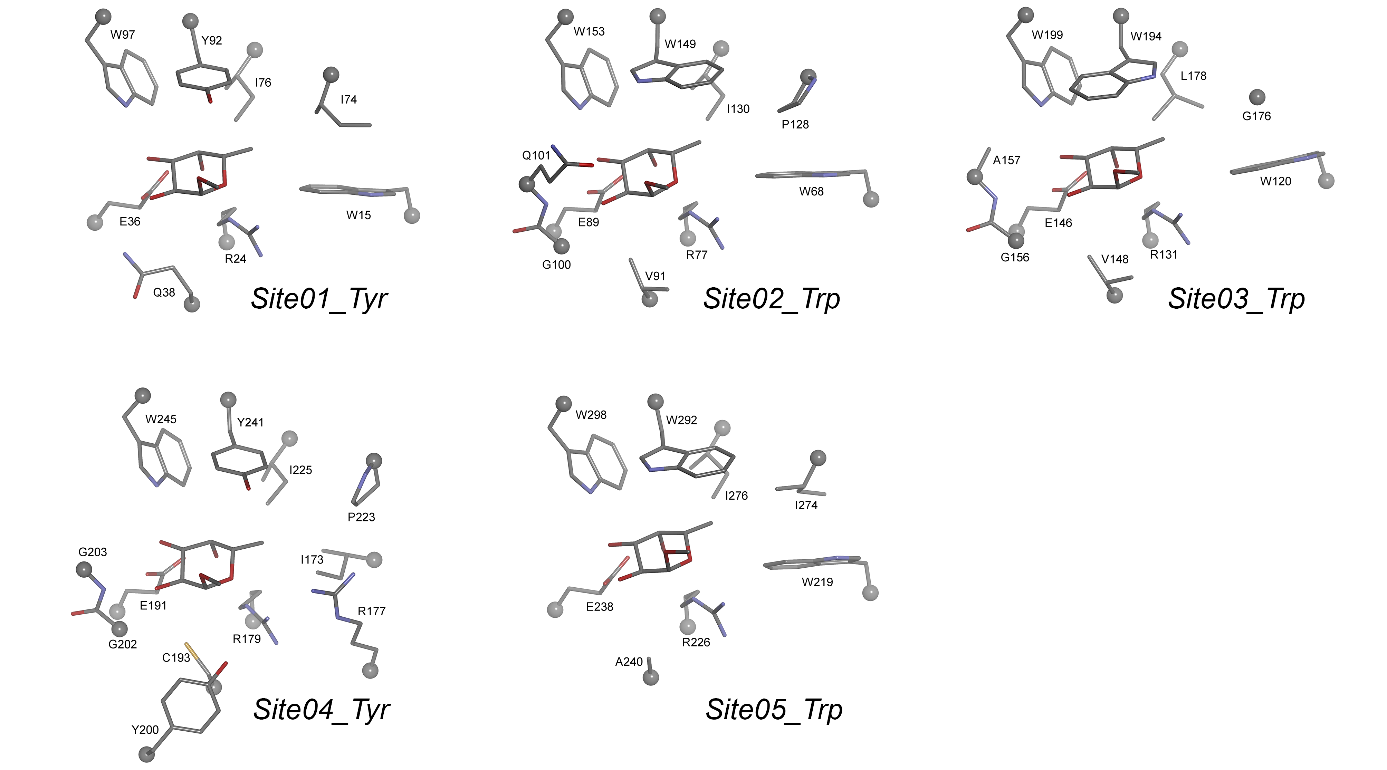

Supplement: S1 Fig — Definition of the AAL binding site models. Fixed CA are shown as a balls. (TIF) [file pone.0189375.s001.tif]

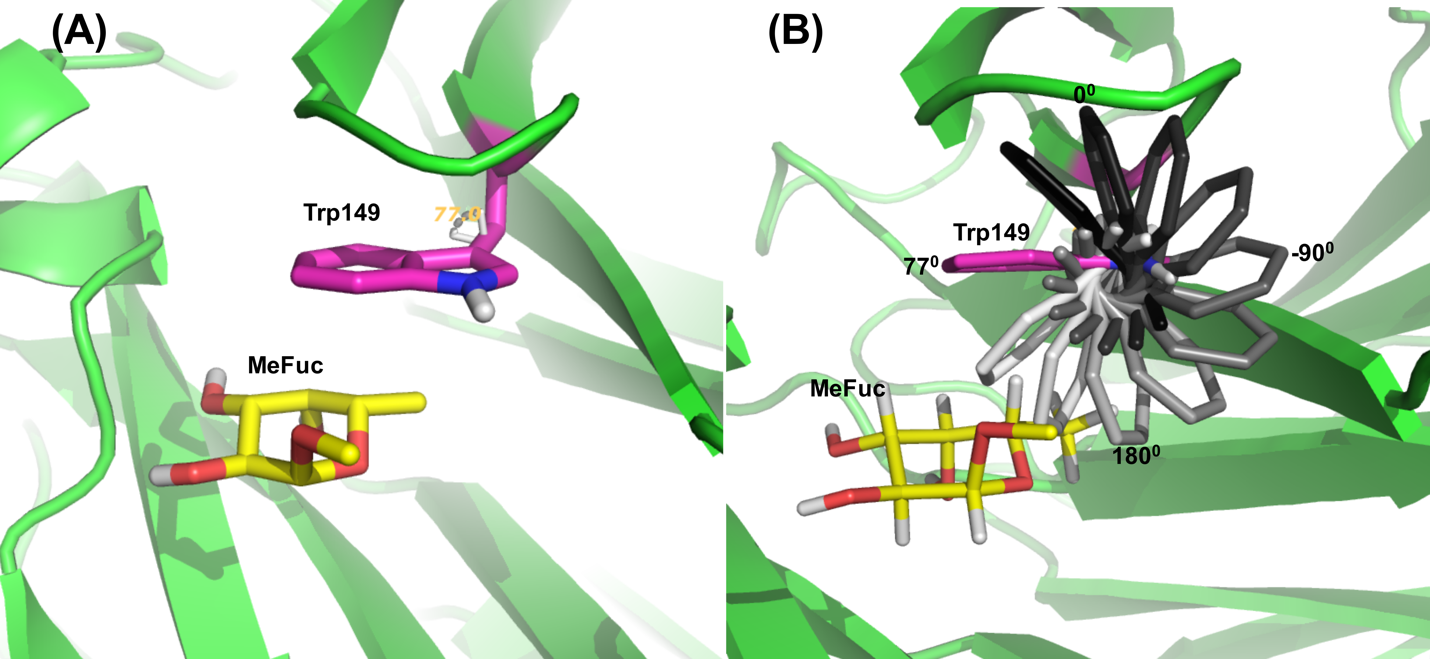

Supplement: S2 Fig — Trp149 interaction with αMeFuc in the binding Site2 (A). Umbrella sampling starting orientations of Trp149 in ligand bound state (B). (TIF) [file pone.0189375.s002.tif]

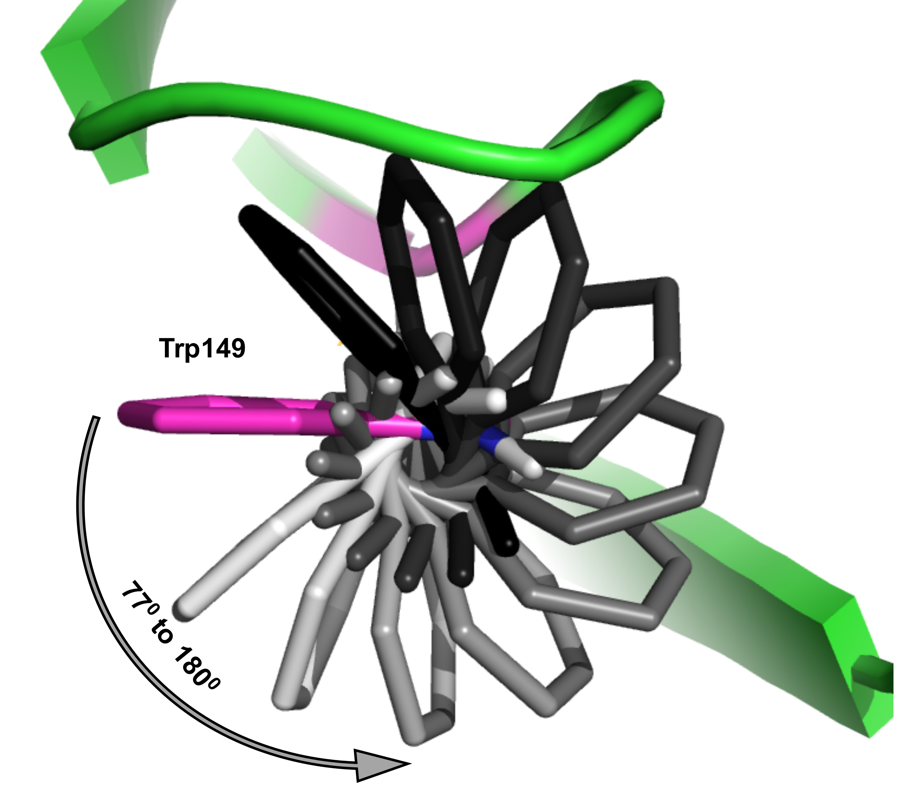

Supplement: S3 Fig — Orientation of Trp149 along the CA-CB-CG-CD2 dihedral angle. (TIF) [file pone.0189375.s003.tif]

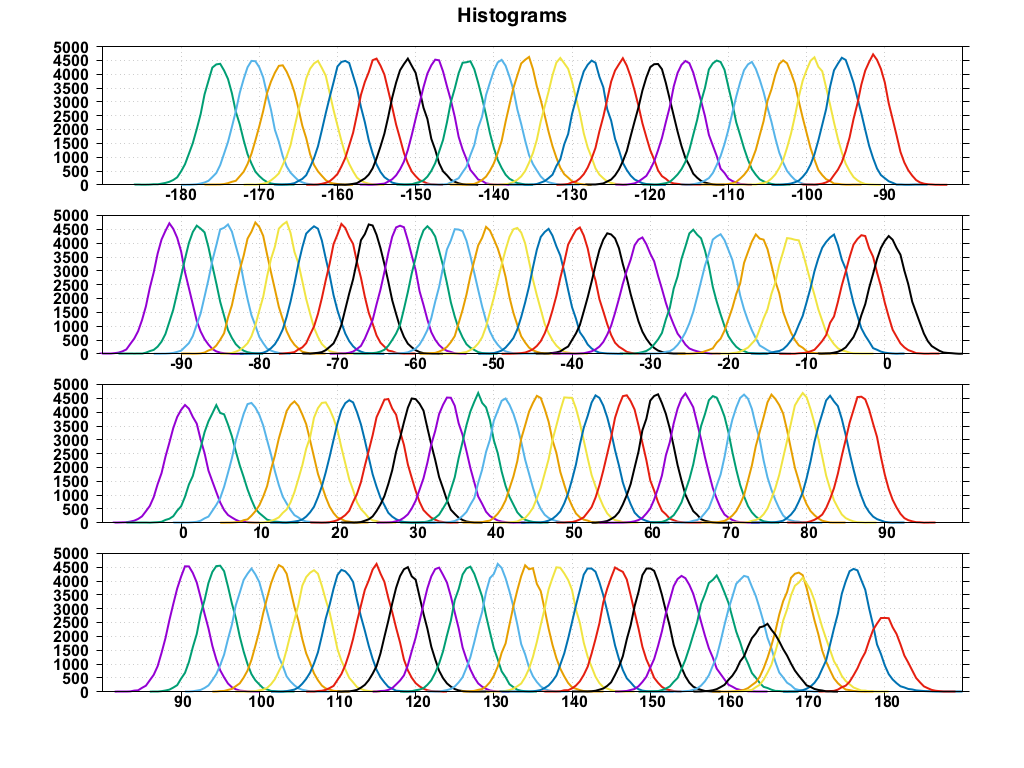

Supplement: S4 Fig — The number of counts (x-axis) for the umbrella sampling histogram at each Ø values in Trp149 (free) state flipping simulation. The overlapped Gaussian-shape histograms confirm the full sampling of whole space -180 to 180 degree. (PNG) [file pone.0189375.s004.png]

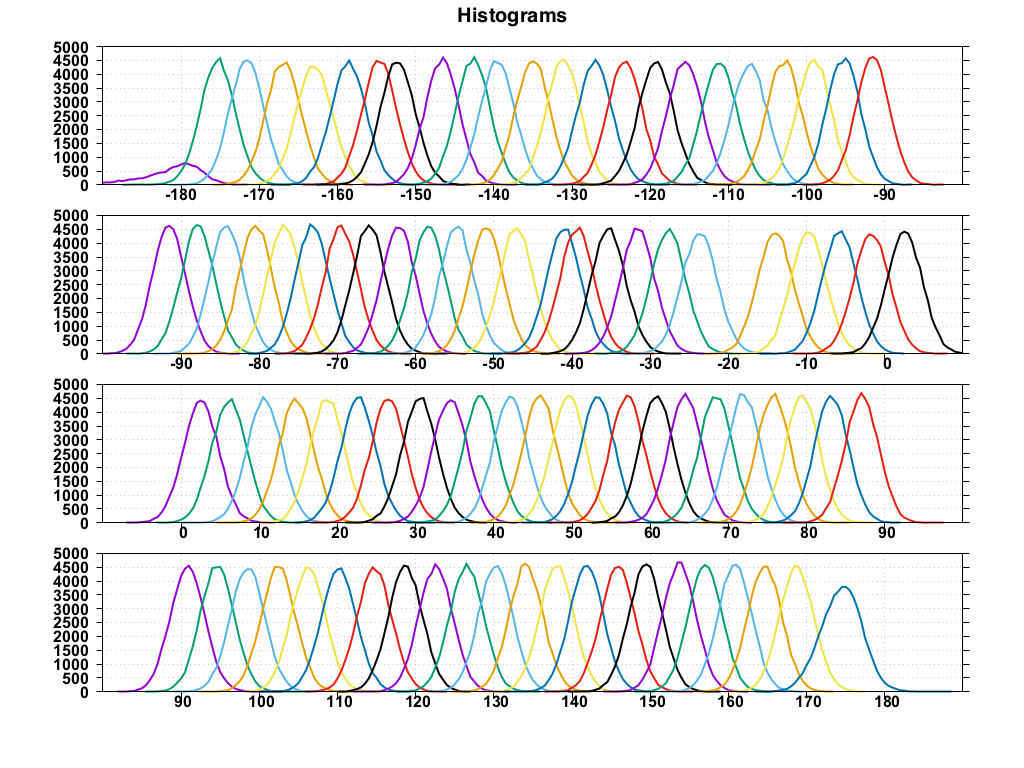

Supplement: S5 Fig — The number of counts (x-axis) for the umbrella sampling histogram at each Ø values in Trp149 (bound) state flipping simulation. The overlapped Gaussian-shape histograms confirm the full sampling of whole space -180 to 180 degree. (PNG) [file pone.0189375.s005.png]

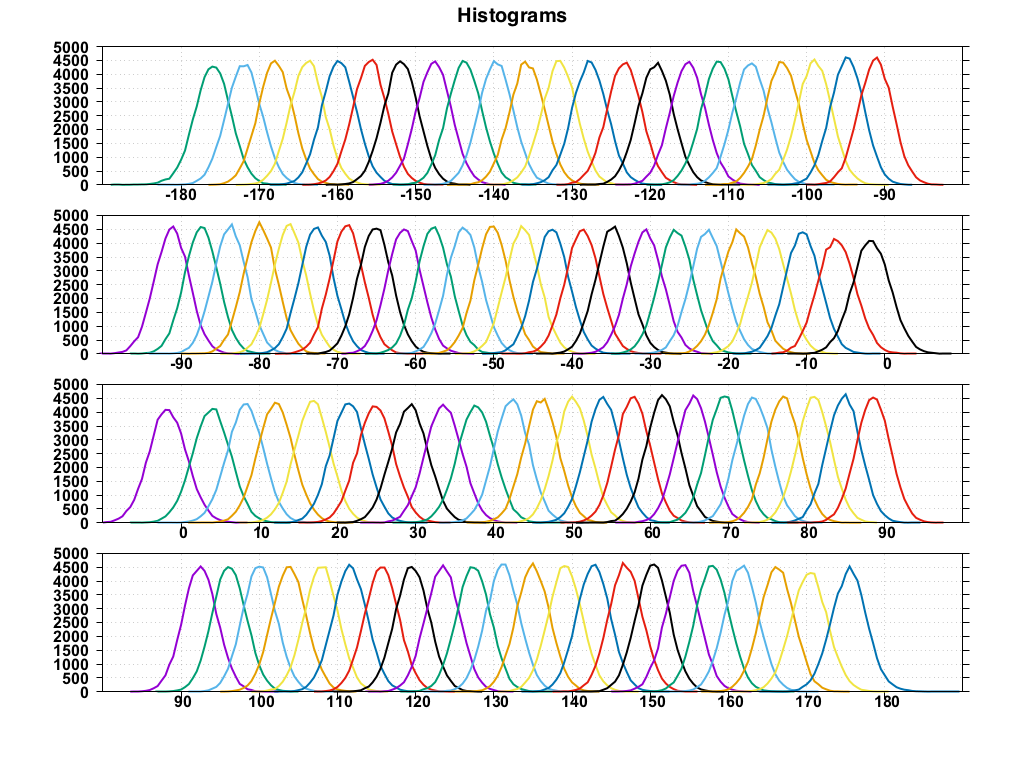

Supplement: S6 Fig — The number of counts (x-axis) for the umbrella sampling histogram at each Ø values in Trp194 (free) state flipping simulation. The overlapped Gaussian-shape histograms confirm the full sampling of whole space -180 to 180 degree. (PNG) [file pone.0189375.s006.png]

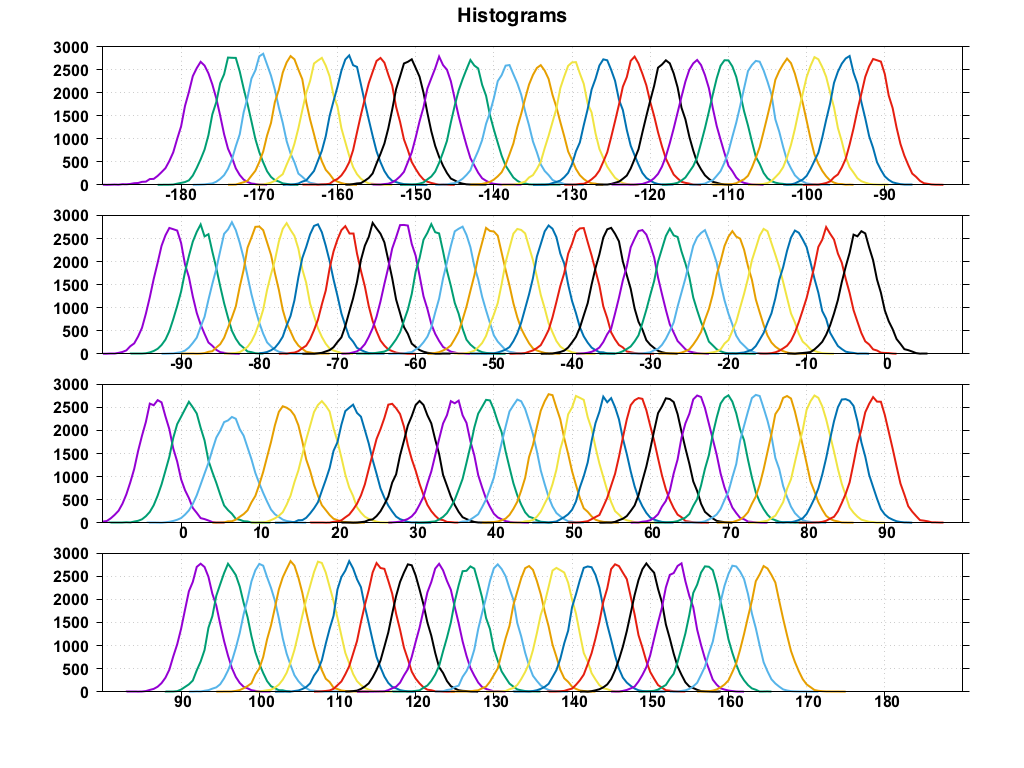

Supplement: S7 Fig — The number of counts (x-axis) for the umbrella sampling histogram at each Ø values in Trp194 (bound) flipping simulations. The overlapped Gaussian-shape histograms confirm the full sampling of whole space -180 to 180 degree. (PNG) [file pone.0189375.s007.png]

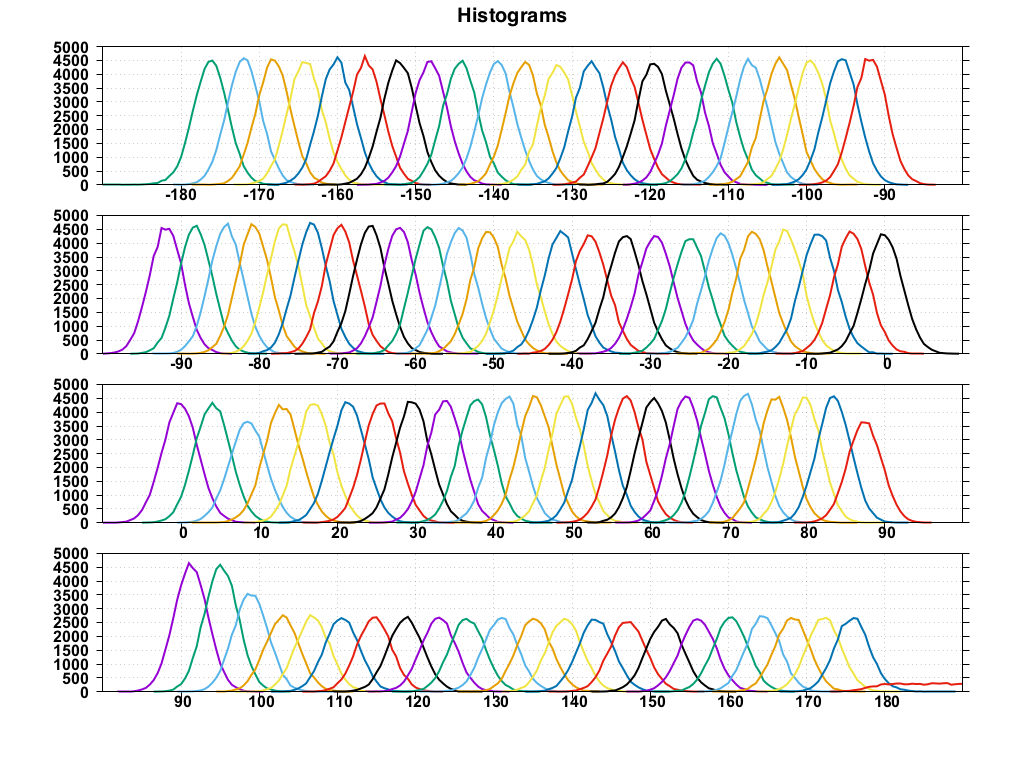

Supplement: S8 Fig — The number of counts (x-axis) for the umbrella sampling histogram at each Ø values in Trp292 (free) flipping simulations. The overlapped Gaussian-shape histograms confirm the full sampling of whole space -180 to 180 degree. (PNG) [file pone.0189375.s008.png]

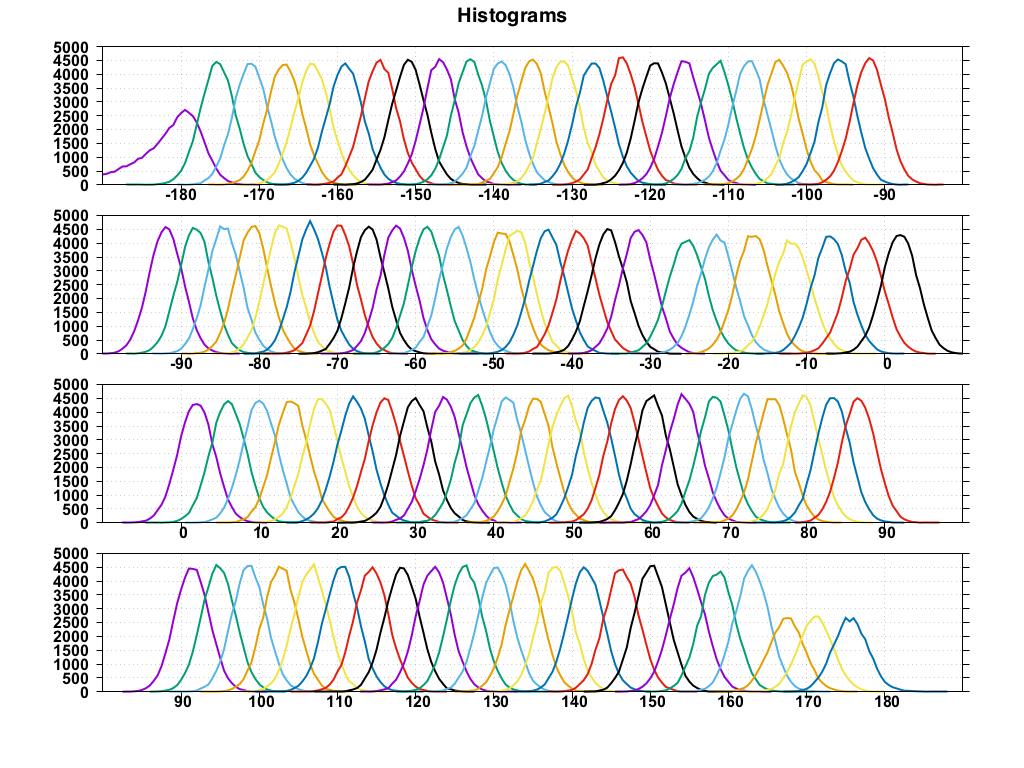

Supplement: S9 Fig — The number of counts (x-axis) for the umbrella sampling histogram at each Ø values in Trp292 (bound) state flipping simulation. The overlapped Gaussian-shape histograms confirm the full sampling of whole space -180 to 180 degree. (PNG) [file pone.0189375.s009.png]
