# Supplementary material for: Influence of Trp flipping on carbohydrate binding in lectins. An example on Aleuria aurantia lectin AAL
Source: PLoS One. 2017 Dec 12;12(12):e0189375. doi: 10.1371/journal.pone.0189375 (PMC5726637; doi:10.1371/journal.pone.0189375)
Supplement: S1 Table — (PDF) [file pone.0189375.s010.pdf]

S1 Table: CH-π stacking Trp conformation in lectins from AAL family.

Protein, PDB, (chain)	Resolution	Site 1	Site 2	Site 3	Site 4	Site 5	Site 6
AAL N224Q 5mxc (A)	1.14	Tyr	*g(+)/g(–)*	*g(–)*	Tyr	*g(+)/g(–)*	NP
AAL 1ofz (A)	1.50	Tyr	*g(–)*	*g(–)*	Tyr	*g(–)*	NP
AAL 1ofz (B)	1.50	Tyr	*g(+)*	*g(–)*	Tyr	*g(–)*	NP
AAL 1iub (A)	2.31	Tyr	*g(+)*	*g(–) UO*	Tyr UO	*g(–) UO*	NP
AAL 1iuc (A)	2.24	Tyr	*g(+)*	*g(–) UO*	Tyr	*g(–) UO*	NP
AFL 4agi (A)	1.60	Tyr	*g(–)*	Tyr	*g(–)*	*g(+)*	Tyr
AFL 4agi (B)	1.60	Tyr	*g(–)*	Tyr	*g(–)*	*g(+)*	Tyr
AFL 4agi (C)	1.60	Tyr	*g(–)*	Tyr	*g(–)*	*g(+)*	Tyr
AFL 4agi (D)	1.60	Tyr	*g(–)*	Tyr	*g(–)*	*g(+)*	Tyr
AFL 4agt (A)	2.00	Tyr	*g(–)*	Tyr	*g(–)*	*g(+)*	Tyr
AFL 4agt (B)	2.00	Tyr	*g(–)*	Tyr	*g(–)*	*g(+)*	Tyr
AFL 4aha (A)	2.20	Tyr	*g(–)*	Tyr	*g(–)*	*g(+)*	Tyr
AFL 4aha (B)	2.20	Tyr	*g(–)*	Tyr	*g(–)*	*g(+)*	Tyr
AFL 4ah4 (A)	1.75	Tyr	*g(–)*	Tyr	*g(–)*	*g(+)*	Tyr
AFL 4ah4 (B)	1.75	Tyr	*g(–)*	Tyr	*g(–)*	*g(+)*	Tyr
AFL 4c1y (A)	2.23	Tyr	*g(–) UO*	Tyr	*g(–) UO*	*g(+) UO*	Tyr
AFL 4c1y (B)	2.23	Tyr	*g(–) UO*	Tyr	*g(–) UO*	*g(+) UO*	Tyr
AFL 4c1y (C)	2.23	Tyr	*g(–) UO*	Tyr	*g(–) UO*	*g(+)*	Tyr
AFL 4c1y (D)	2.23	Tyr UO	*g(+) UO*	Tyr	*g(–) UO*	*g(+) UO*	Tyr
AFL 4uou (A)	2.40	Tyr UO	*g(+) UO*	Tyr UO	*g(–) UO*	*g(+) UO*	Tyr UO
AFL 4uou (B)	2.40	Tyr UO	*g(+) UO*	Tyr UO	*g(–) UO*	*g(+) UO*	Tyr UO
AFL 4uou (C)	2.40	Tyr UO	*g(–) UO*	Tyr UO	*g(–) UO*	*g(+) UO*	Tyr UO
AFL 4uou (D)	2.40	Tyr UO	*g(+) UO*	Tyr UO	*g(–) UO*	*g(+) UO*	Tyr UO
AFL 4d52 (A)	1.76	Tyr	*g(–)*	Tyr	*g(–) UO*	*g(+) UO*	Tyr
AFL 4d52 (B)	1.76	Tyr	*g(–)*	Tyr	*g(–) UO*	*g(+) UO*	Tyr
AFL 4d52 (C)	1.76	Tyr	*g(–)*	Tyr	*g(–) UO*	*g(+) UO*	Tyr

AFL 4d52 (D)	1.76	Tyr	*g(–)*	Tyr	*g(–) UO*	*g(+) UO*	Tyr
AFL 4d4u (A)	1.99	Tyr	*g(–)*	Tyr	*g(–) UO*	*g(+)*	Tyr
AFL 4d4u (B)	1.99	Tyr	*g(–)*	Tyr	*g(–) UO*	*g(+)*	Tyr
RSL 2bs5 (A)	2.10	*g(–)*	*g(–)*	*g(–) sym*	*g(–) sym*	*g(–) sym*	*g(–) sym*
RSL 2bs6 (A)	1.80	*g(–)*	*g(–)*	*g(–) sym*	*g(–) sym*	*g(–) sym*	*g(–) sym*
RSL 2bt9 (A, B, C)	0.94	*g(–)*	*g(–)*	*g(–)*	*g(–)*	*g(–)*	*g(–)*
RSL R17A 3zi8 (A, B, C)	1.50	*g(–) UO*	*g(–)*	*g(–) sym UO*	*g(–) sym*	*g(–) sym UO*	*g(–) sym*
RSL W76A 4i6s (A, B, C)	1.54	Ala	*g(–)*	Ala	*g(–)*	Ala	*g(–)*
RSL mono 4csd (A)	1.35	*g(–)*	*g(–)*	*g(–)*	*g(–)*	*g(–)*	*g(–)*
RSL mono 4csd (B)	1.35	*g(–)*	*g(–)*	*g(–)*	*g(–)*	*g(–)*	*g(–)*
RSL 5ajb (A, B, C)	1.80	*g(–)*	*g(–)*	*g(–)*	*g(–)*	*g(–)*	*g(–)*
RSL 5ajc (A, B, C)	1.70	*g(–)*	*g(–)*	*g(–)*	*g(–)*	*g(–)*	*g(–)*
BambL 3zw0 (A, B, C)	1.60	*g(–) UO*	Tyr UO	*g(–) UO*	Tyr UO	*g(–)*	Tyr UO
BambL 3zwe (A, B, C)	1.75	*g(–)*	Tyr	*g(–)*	Tyr	*g(–)*	Tyr
BambL 3zzv (A, B, C)	1.68	*g(–)*	Tyr	*g(–)*	Tyr	*g(–)*	Tyr
BambL 3zw1 (A)	1.60	*g(–)*	Tyr	*g(–) sym*	Tyr sym	*g(–) sym*	Tyr sym
BambL 3zw1 (B)	1.60	*g(–)*	Tyr	*g(–) sym*	Tyr sym	*g(–) sym*	Tyr sym
BambL 3zw2 (A, B, C)	1.60	*g(–)*	Tyr	*g(–)*	Tyr	*g(–)*	Tyr

Tyr – tyrosine instead of tryptophan residue, Ala – alanine instead of tryptophan residue, NP – site not present, UO – site not occupied by sugar ligand, sym – site exists as symmetry mate to another site in propeller (for bacterial trimeric lectins RSL and BambL)
