# Supplementary material for: Influence of Trp flipping on carbohydrate binding in lectins. An example on Aleuria aurantia lectin AAL
Source: PLoS One. 2017 Dec 12;12(12):e0189375. doi: 10.1371/journal.pone.0189375 (PMC5726637; doi:10.1371/journal.pone.0189375)

S2 Table: Distribution of the Trp conformation in observed carbohydrate – Trp complexes from PDB database.

Conformation	Number of Complexes
trans (-180° +30°)	3
gauche (-) (-90° ±60°)	2083
eclipsed (0° ±30°)	581
gauche (+) (90° ±60°)	2526
trans (180° -30°)	15

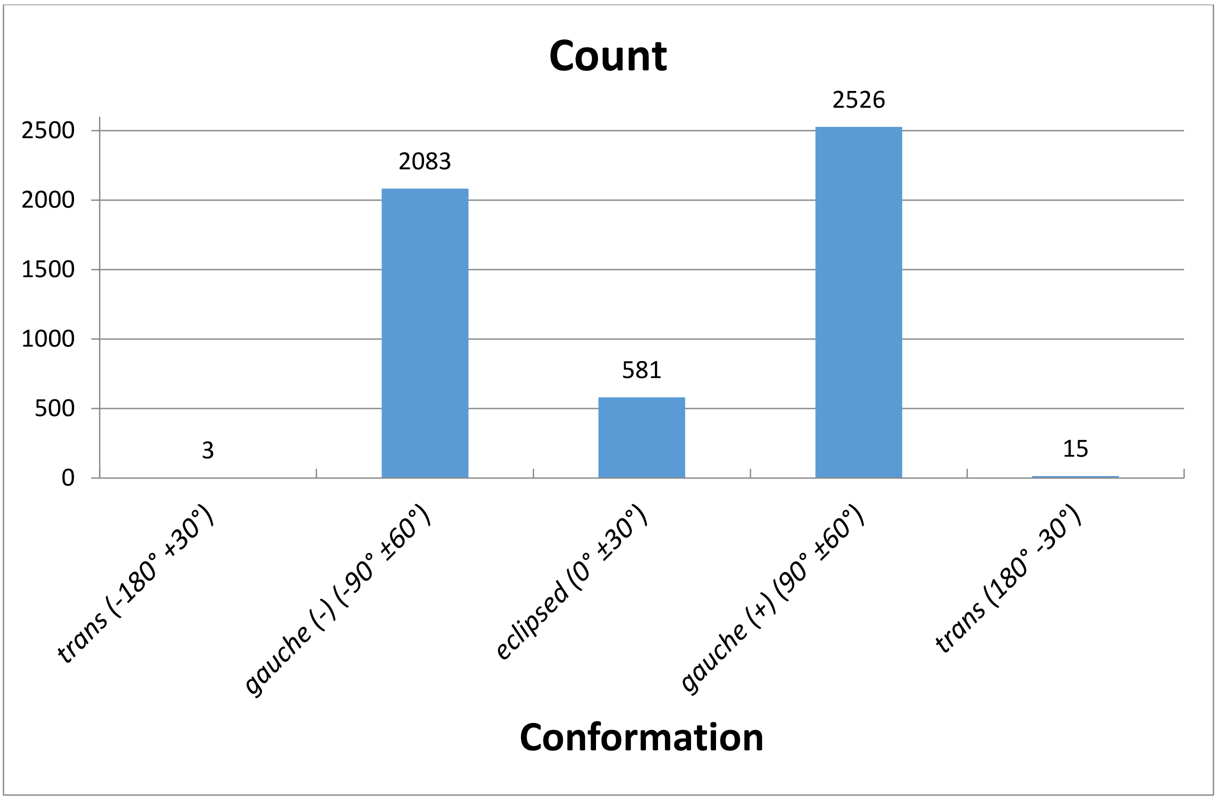

Supplement: S2 Table — (PDF) [file pone.0189375.s011.pdf]
