# Supplementary material for: Influence of Trp flipping on carbohydrate binding in lectins. An example on Aleuria aurantia lectin AAL
Source: PLoS One. 2017 Dec 12;12(12):e0189375. doi: 10.1371/journal.pone.0189375 (PMC5726637; doi:10.1371/journal.pone.0189375)
Supplement: S1 File — Detailed information about used protocol for Umbrella Sampling MD. (PDF) [file pone.0189375.s012.pdf]

Equilibration protocol for Umbrella Sampling MD:

First of all, the solvent molecules were energy minimized over 3000 steps of steepest descent while keeping the solute atoms restrained with a force constant of 50 kcal/mol restraint. The simulation box was then heated to 300 K during a 100 ps long NVT simulation subsequently followed by a 300-ps long NPT simulation where solute atoms were subjected to 50 kcal/mol restraint. Thereafter, a series of NPT simulations (10 ps, 10 ps, 50 ps, 50 ps, 70 ps) were performed, where restraint was decreased slowly (25, 10, 5, 2.5, 1 kcal/mol, respectively) to relax the whole system. This was followed by a 300 ps NPT simulation where only backbone atoms (C, CA, O, N) were restrained by a 1 kcal/mol restraint. Finally, all the restrains were removed and a 300-ps NPT simulation was carried out to equilibrate the whole system.
