# Supplementary material for: Influence of Trp flipping on carbohydrate binding in lectins. An example on Aleuria aurantia lectin AAL
Source: PLoS One. 2017 Dec 12;12(12):e0189375. doi: 10.1371/journal.pone.0189375 (PMC5726637; doi:10.1371/journal.pone.0189375)
Supplement: S2 File — Description of the methodology used for the PDB data mining. (PDF) [file pone.0189375.s013.pdf]

PDB database data mining details

PDB database was searched to find binding sites with sugar ligands that are bound by CH-π stacking interaction with tryptophan. We used PatternQuery program for searching. (https://webchem.ncbr.muni.cz/Platform/PatternQuery)

Sugar ligand was defined as a ligand that contains a ring without multiple bonds that consists of one oxygen atom and four or five carbon atoms and have OH group bound to C3 or C4 ring atom (atom names are based on PDB database nomenclature).

CH-π stacking interaction was defined using distance between Trp and sugar ligand up to 4.5Å and the size of torsion angle between Trp and sugar ligand 45 – 135° (cut off values are not included).

The distance was measured between the aromatic centre of Trp (the centre of CD2, CE2 bond) and the closest CH atom of ligand. Only CH atoms inside the ring or bound to a ring atom were considered.

The torsion angle between Trp and sugar ligand was defined as an angle between NE1 – CE2 – CD2 Trp atoms and the closest CH atom of ligand (the same CH atom was used for distance measurement).

Hydrogen atoms were added to structures using OpenBabel 2.3.90 software package.

In found motives with CH-π stacking interaction we looked at the torsion angle of Trp between atoms CA – CB – CG – CD1.
